# Generation and Proteome Profiling of PBMC-Originated, iPSC-Derived Corneal Endothelial Cells

**DOI:** 10.1167/iovs.17-22927

**Published:** 2018-05

**Authors:** Muhammad Ali, Shahid Y. Khan, Shivakumar Vasanth, Mariya R. Ahmed, Ruiqiang Chen, Chan Hyun Na, Jason J. Thomson, Caihong Qiu, John D. Gottsch, S. Amer Riazuddin

**Affiliations:** 1 The Wilmer Eye Institute, Johns Hopkins University School of Medicine, Baltimore, Maryland, United States; 2 Department of Biological Chemistry, Johns Hopkins University School of Medicine, Baltimore, Maryland, United States; 3 Yale Stem Cell Center, Yale University School of Medicine, New Haven, Connecticut, United States

**Keywords:** corneal endothelial cells, induced pluripotent stem cells, mass-spectrometry–based proteome sequencing

## Abstract

**Purpose:**

Corneal endothelial cells (CECs) are critical in maintaining clarity of the cornea. This study was initiated to develop peripheral blood mononuclear cell (PBMC)-originated, induced pluripotent stem cell (iPSC)-derived CECs.

**Methods:**

We isolated PBMCs and programmed the mononuclear cells to generate iPSCs, which were differentiated to CECs through the neural crest cells (NCCs). The morphology of differentiating iPSCs was examined at regular intervals by phase contrast microscopy. In parallel, the expression of pluripotent and corneal endothelium (CE)-associated markers was investigated by quantitative real-time PCR (qRT-PCR). The molecular architecture of the iPSC-derived CECs and human corneal endothelium (hCE) was examined by mass spectrometry–based proteome sequencing.

**Results:**

The PBMC-originated, iPSC-derived CECs were tightly adherent, exhibiting a hexagonal-like shape, one of the cardinal characteristics of CECs. The CE-associated markers expressed at significantly higher levels in iPSC-derived CECs at days 13, 20, and 30 compared with their respective levels in iPSCs. It is of importance that only residual expression levels of pluripotency markers were detected in iPSC-derived CECs. Cryopreservation of iPSC-derived CECs did not affect the tight adherence of CECs and their hexagonal-like shape while expressing high levels of CE-associated markers. Mass spectrometry–based proteome sequencing identified 10,575 proteins in the iPSC-derived CEC proteome. In parallel, we completed proteome profiling of the hCE identifying 6345 proteins. Of these, 5763 proteins were identified in the iPSC-derived CECs, suggesting that 90.82% of the hCE proteome overlaps with the iPSC-derived CEC proteome.

**Conclusions:**

We have successfully developed a personalized approach to generate CECs that closely mimic the molecular architecture of the hCE. To the best of our knowledge, this is the first report describing the development of PBMC-originated, iPSC-derived CECs.

The cornea is the outermost, transparent layer of the eye. It is composed of five layers: epithelium, Bowman's membrane, stroma, Descemets membrane, and endothelium.^1^ The corneal endothelium (CE) is a monolayer of hexagonal cells that is critical in maintaining corneal clarity by mediating hydration through barrier and pump functions.^2^ The CE cell density is approximately 2500 cells/mm^2^ in adult CE.^3^ Corneal endothelial dystrophies and surgical trauma are the major factors that contribute to loss of corneal endothelial cells (CECs) and a decrease in CE cell density. The physiological functioning of the CE is undermined below 500 cells/mm^2^ cell density, resulting in corneal edema and loss of vision.^3^

Fuchs corneal endothelial dystrophy (FCD) is the leading cause of corneal transplantation performed both in the United States and worldwide each year.^4,5^ Although keratoplasty has been successful at visual rehabilitation, graft rejection and lack of suitable donor tissue for transplantation continue to be impediments to reduce worldwide corneal blindness. CECs have been generated previously from human pluripotent stem cells.^6,7^ Fukuta and colleagues^8^ differentiated human pluripotent stem cells into CECs using chemically defined conditions. Recently, McCabe and colleagues^9^ generated CECs from embryonic stem cells through the neural crest cells (NCCs).^9^

Here, we report derivation of CECs from peripheral blood mononuclear cell (PBMC)-originated, induced pluripotent stem cells (iPSCs). We characterized the proteome of the iPSC-derived CECs through mass spectrometry–based protein sequencing and subsequently compared it with the proteome of a CE specimen obtained from an eye bank. This analysis suggested that >90% of the proteins expressed in donor CE are present in iPSC-derived CECs. To the best of our knowledge, this is the first report of PBMC-originated, iPSC-derived CECs.

## Materials and Methods

### Subjects and Clinical Ascertainment

Institutional review board approvals for research involving human subjects and pluripotent stem cells were obtained from the Johns Hopkins University School of Medicine (Baltimore, MD, USA). The control participating subject gave informed consent consistent with the tenets of the Declaration of Helsinki and underwent a thorough ophthalmic examination to exclude any ocular anomaly.

### Isolation and Cryopreservation of PBMCs

The PBMCs were isolated according to the procedure published by Agu and colleagues.^10^ Briefly, 10 mL human peripheral blood was collected in a 50 mL conical tube containing 0.5 M EDTA, and two volumes of phosphate-buffered saline (PBS) was added. The mixture was gently layered on 15 mL histopaque (MilliporeSigma, Burlington, MA, USA) and centrifuged at 400*g* for 30 minutes (without brakes).

The mononuclear cells layer was gently collected in 50 mL tube and washed twice with PBS by centrifugation at 300*g* for 10 minutes. The PBMC pellet was cryopreserved in 10% dimethyl sulfoxide (DMSO; MilliporeSigma) supplemented with 90% fetal bovine serum (MilliporeSigma).

### Human Embryonic Stem Cells (hESCs)

The H9 hESCs (WiCell Research Institute, Madison, WI, USA) were cultured in mTeSR1 medium (STEMCELL Technologies, Inc., Vancouver, BC, Canada) in feeder-free conditions on Matrigel-coated plates (Corning, Bedford, MA, USA). The cells were passaged using Accutase (MilliporeSigma) every 5 to 6 days, and culture medium was changed daily.

### Human Corneal Endothelium (hCE)

Keratoplasty-grade donor corneas (>55 years of age) from postmortem eyes were obtained from Tissue Bank International (Baltimore, MD, USA) for expression analysis of hCE-associated markers. The donor corneas were preserved and transported in Optisol-GS (Bausch & Lomb, Rochester, NY, USA) at 4°C, and were used within 3 days of preservation. The corneoscleral tissues were washed twice with PBS. The hCE was carefully peeled off the cornea under a dissection microscope (Thermo Fisher Scientific, Waltham, MA, USA) and stored at −70°C until further use.

### Generation of iPSCs Using the Sendai Virus Delivery System

The cryopreserved PBMCs were reprogramed using a Sendai virus delivery system kit, according to the manufacturer's instructions (Cytotune 2.0; Life Technologies, Carlsbad, CA, USA). Briefly, the PBMC vial was removed from liquid nitrogen and thawed at 37°C in a water bath. Subsequently, the PBMCs were washed with medium (StemSpan; STEMCELL Technologies, Inc.) and cultured in StemSpan medium supplemented with 100 ng/mL Feline McDonough Sarcoma-like tyrosine kinase 3 ligand (FLT-3L), 100 ng/mL stem cell factor, 20 ng/mL interleukin-3 (IL-3), and 20 ng/mL interleukin-6 (IL-6), termed complete medium hereafter, in a humidified incubator at 37°C supplemented with 5% CO_2_ for 4 days.

Approximately, 5 × 10^5^ cells/mL PBMCs were collected in a round-bottom tube in 1 mL complete medium, followed by infection by reprogramming viral particles at a multiplicity of infection (MOI) of 5, 5, and 3 (KOS MOI = 5; hc-MYC MOI = 5; hKLF4 MOI = 3). The infected cells were centrifuged at 1000*g* for 30 minutes at room temperature. The cells were resuspended in 1 mL complete medium, transferred to a single well of a 12-well plate, and cultured for 3 days in complete medium. Subsequently, the cells were transferred to mouse embryonic fibroblast–coated plates for 3 days with complete medium and finally transferred to Dulbecco's modified Eagle's medium-F12 (DMEM-F12) supplemented with 20% knockout serum replacement (KSR; Life Technologies) and 20 ng/mL basic fibroblast growth factor (bFGF; Life Technologies) until an embryonic stem cell–like colony was formed. Embryonic stem cell–like putative iPSC colonies were selected and cultured on a Matrigel-coated (Corning) plate in mTeSR1 medium for characterization.

### Phase Contrast Microscopy

Phase contrast microscopy was performed using an inverted microscope (Zeiss Axio Observer A1; Carl Zeiss, Jena, Germany) equipped with imaging software (Q-Capture; QImaging, Surrey, BC, Canada).

### Characterization of iPSCs by Flow Cytometry

The PBMC-originated, iPSCs were characterized by flow cytometry. Approximately 2 × 10^5^ cells were labeled with SSEA4 and TRA-1-60 (Cell Signaling Technology, Danvers, MA, USA) primary antibodies for 1 hour at 4°C followed by treatment with FITC-conjugated goat anti-mouse IgG antibody (against SSEA4 and TRA-1-60; MilliporeSigma) for 40 minutes at 4°C. A total of 10,000 events were acquired for each sample using a flow cytometer (Guava easyCyte; MilliporeSigma).

### Characterization of iPSCs by Quantitative Real-Time Polymerase Chain Reaction (qRT-PCR)

The total RNA from H9 hESCs and the PBMC-originated, iPSCs was extracted using reagent (TRIzol; Invitrogen; Carlsbad, CA, USA) according to the manufacturer's instructions. The RNA was quantitated on a spectrophotometer (NanoDrop Lite; Thermo Fisher Scientific). First-strand cDNA was synthesized using a kit (Superscript III; Invitrogen) according to the manufacturer's instructions.

qRT-PCR was performed on the STEP ONE ABI Real-Time PCR System (Applied Biosystems, Foster City, CA, USA). The expression of pluripotent markers (*NANOG, OCT4, SOX2,* and *TRA-1-60*) were quantitated using Power SYBR Green PCR Master Mix (Life Technologies). *GAPDH* was used as the endogenous control. The delta–delta Ct method was used to determine the relative expression, normalized against *GAPDH*, as reported previously.^11,12^ All the primers used in the qRT-PCR analysis were designed using a real-time PCR tool (Integrated DNA Technologies, Coralville, IA, USA) and are available upon request.

### Differentiation of iPSCs Into CECs

We used PBMC-originated, iPSCs to generate CECs by modifying a previously published procedure.^9^ Briefly, iPSCs were seeded on 35-mm Matrigel-coated plates (Corning) in 1:12 dilution (80% confluent plate was split into 12 plates) on day 0 using cell dissociation buffer (Life Technologies). The iPSCs were grown for 4 days in medium (mTeSR1; STEMCELL Technologies, Inc.). On day 4, mTeSR1 media was replaced with dual Smad inhibitors media containing 500 ng/mL human recombinant Noggin (R&D Systems, Minneapolis, MN, USA) and 10 μM SB431542 (MilliporeSigma) in a basal media of 80% DMEM-F12 (Life Technologies), 20% KSR (Life Technologies), 1% nonessential amino acids (Life Technologies), 1 mM l-glutamine (STEMCELL Technologies, Inc.), 0.1 mM β-mercaptoethanol (MilliporeSigma), and 8 ng/mL bFGF (MilliporeSigma).

On day 6, dual Smad inhibitors media was replaced by cornea medium containing 0.1× B27 supplement (Life Technologies), 10 ng/mL recombinant human platelet derived growth factor-BB (PDGF-BB; PeproTech, Rocky Hill, NJ, USA), and 10 ng/mL recombinant human Dickkopf related protein-2 (DKK-2; R&D Systems) in a basal media of 80% DMEM-F12 (Life Technologies), 20% KSR (Life Technologies), 1% nonessential amino acids (Life Technologies), 1 mM l-glutamine (STEMCELL Technologies, Inc.), 0.1 mM β-mercaptoethanol (MilliporeSigma), and 8 ng/mL bFGF (MilliporeSigma). On day 7, the differentiating CECs were transferred to new Matrigel-coated plates (35 mm) and were grown in cornea medium for 13 additional days. The differentiated CECs were harvested on day 20 for mass spectrometry–based proteome sequencing.

### Characterization of iPSC-Derived CECs by qRT-PCR

The total RNA from H9 hESCs, PBMC-originated iPSCs, iPSC-derived differentiating CECs at days 6, 13, 20, and 30 and hCE was extracted using TRIzol reagent (Invitrogen). The first-strand cDNA was synthesized using the Superscript III kit (Invitrogen) as described above. The expression of pluripotent markers (*NANOG, OCT4, SOX2,* and *TRA-1-60*), neural crest markers (*NGFR*, *SOX10*), and CE-associated markers (*AQP1, COL4A1, COL4A3, COL8A1, COL8A2, FOXC1,* and *SLC16A3*) were quantitated using qRT-PCR as described above. The primers were designed using a real-time PCR tool (Integrated DNA Technologies) and are available upon request.

### Cryopreservation of iPSC-Derived CECs and qRT-PCR Analysis

The differentiated CECs at day 20 were detached using cell dissociation buffer (Life Technologies) and resuspended in cornea medium. The cells were collected by centrifugation and cryopreserved in freezing medium comprising 90% cornea medium and 10% DMSO. The cryopreserved iPSC-derived CECs were cultured a week later and maintained in cornea medium until they reached confluence. The expression of CE-associated markers (*AQP1, ATP1A1, ATP1A3, COL4A1*, *COL4A3, COL8A1, COL8A2, FOXC1,* and *SLC16A3*) was quantitated using qRT-PCR as described above.

### Characterization of Cryopreserved iPSC-Derived CECs by Immunocytochemistry

The cryopreserved iPSC-derived CECs were also analyzed by immunocytochemistry. Briefly, the cells were fixed with 4% paraformaldehyde (PFA) for 15 minutes followed by blocking with 5% bovine serum albumin (MilliporeSigma). The cells were first incubated with 1:100 zona occludens-1 (ZO-1; Cell Signaling Technology), 1:50 N-cadherin (Cell Signaling Technology), and 1:40 ATPase sodium/potassium subunit alpha1 (Na^+^/K^+^ATPase α1; Santa Cruz Biotechnology) primary antibodies overnight at 4°C. The cells were next treated with 1:100 FITC-conjugated goat anti-rabbit IgG (against ZO-1; MillporeSigma), 1:75 FITC-conjugated goat anti-mouse IgG (against N-cadherin; MilliporeSigma), and 1:75 FITC-conjugated goat anti-mouse IgG (against Na^+^/K^+^ATPase α1; MilliporeSigma) secondary antibodies for 2 hours at room temperature. The nuclei were counterstained with 4′,6-diamidine-2′-phenylindole dihydrochloride (DAPI; MilliporeSigma). The images of mounted cells were captured using a microscope (Olympus LX81; Olympus, Tokyo, Japan) equipped with software (Slidebook Software 3i; Denver, CO, USA) and prepared using image-editing software (Adobe Photoshop CS5; Adobe Systems, Inc., San Jose, CA, USA).

### Protein Extraction, and Digestion

Human iPSC-derived CECs at day 20 from two 35-mm culture plates were harvested, pooled together, and subjected to a mass spectrometry–based label-free quantitative proteomics. Sample processing including protein isolation, digestion, and analysis on liquid chromatography-mass spectrometry was performed with little modifications as described previously.^12^ Briefly, the CECs were lysed by sonicating in 8 M urea and 50 mM triethylammonium bicarbonate. Protein lysates were centrifuged at 16,000*g* at 4°C to exclude cell debris (pelleting at the bottom), and protein concentration was estimated using a bicinchoninic acid assay. Total protein sample (2600 μg) was reduced with 10 mM dithiothreitol (MilliporeSigma) at room temperature for 1 hour and alkylated with 30 mM iodoacetamide (MilliporeSigma) for 20 minutes (in dark). The protein sample was digested with LysC (1:100; Wako Chemicals, Inc., Richmond, VA, USA) by incubating at room temperature for 3 hours followed by an overnight digestion at 37 °C using sequencing-grade trypsin (1:50; Promega Corp., Madison, WI, USA) and finally desalted with C18 Sep-Pak (Waters Corp., Milford, MA, USA). The peptides were fractionated by basic pH reversed-phase liquid chromatography (Waters Corp.) into 96 fractions, followed by concatenation into 24 fractions by combining every 24th fraction.

A pair of corneas from a 62-year-old female donor (postmortem) was obtained from Eversight Eye Bank (available in the public domain, http://eversightvision.org) for proteome profiling of hCE. The hCE was carefully peeled off the cornea under a dissection microscope (Thermo Fisher Scientific); nevertheless, it may have contained a residual amount of Descemets membrane. The hCE tissue from both eyes was pooled together, and the total protein sample (210 μg) was subjected to a mass spectrometry–based label-free quantitative proteomics as described above for human iPSC-derived CECs.

### Mass Spectrometry-Based Proteome Sequencing

The fractionated peptides were analyzed on a mass spectrometer coupled with a nano-flow liquid chromatography system (Orbitrap Fusion Lumos Tribrid and EASY-nLC 1200 system, respectively; Thermo Fisher Scientific). The peptides from each fraction were reconstituted in 15 μL 0.1% formic acid and loaded on columns (Acclaim PepMap 100 Nano-Trap, 100 μm × 2 cm; Thermo Fisher Scientific) packed with 5 μm C_18_ particles at a flow rate of 5 μL per minute. Peptides were resolved at 250 nL per minute flow rate using a linear gradient of 10% to 35% solvent B (0.1% formic acid in 95% acetonitrile) over 95 minutes on a column (EASY-Spray, 50 cm × 75 μm; Thermo Fisher Scientific) packed with 2 μm C_18_ particles (Thermo Fisher Scientific), which was fitted with an EASY-Spray ion source operated at a voltage of 2.0 kV.

Mass spectrometry analysis was completed in a data-dependent manner with a full scan in the mass-to-charge ratio (*m/z*) range of 350 to 1800 in the “top speed” setting, 3 seconds per cycle. MS1 was acquired for the precursor ions measured at a resolution of 120,000 at an *m/z* of 200. An MS2 scan was acquired by fragmenting precursor ions using a higher-energy collisional dissociation (HCD) method and detected at a mass resolution of 30,000 at an *m/z* of 200. Automatic gain control for MS1 was set to one million ions and for MS2 was set to 0.05 million ions. A maximum ion injection time was set to 50 milliseconds for MS1 and 100 milliseconds for MS2 (HCD was set to 32 with stepped collision energy of 5% for MS2). Precursor isolation window was set to 2.0 *m/z* with 0.5 *m/z* offset. Dynamic exclusion was set to 30 seconds, and singly charged ions were rejected. Internal calibration was carried out using the lock mass option (*m/z* 445.1200025) from ambient air.

The fractionated peptides for hCE were analyzed on an Orbitrap Fusion Lumos Tribrid mass spectrometer, and mass spectrometry analysis was carried out in a data-dependent manner with a full scan in the *m/z* range as described above for human iPSC-derived CECs.

### Data Analysis

MaxQuant (v1.5.3.8.) software was used for quantitation and identification. During tandem mass spectrometry (MS/MS) preprocessing, the top 12 peaks in each window of 100 *m/z* were selected for database search. The MS/MS data were then searched using Andromeda search algorithms against UniProt (Swiss-Prot only; released April 2017) and neXtprot (released August 2017) databases with common contaminant proteins for iPSC-derived CEC and hCE proteomes, respectively. Search parameters included (1) trypsin as a proteolytic enzyme with up to two missed cleavages; (2) first search peptide mass error tolerance of 20 ppm and the main search peptide mass error tolerance of 4 ppm; (3) fragment mass error tolerance of 20 ppm; (4) carbamidomethylation of cysteine (+57.02146 Da) as a fixed modification; and (5) oxidation of methionine (+15.99492 Da) and protein acetyl (+ 42.01056 Da) on N-terminus as a dynamic modifications. The minimum peptide length was set to seven amino acids, and the minimum number of peptide sequence per protein was set to two peptides. Peptides and proteins were filtered at 1% false-discovery rate whereas the label-free quantification, the iBAQ, and the second peptides parameters were enabled. A minimum ratio count was set to two, and both unique and razor peptides were used for quantification. After protein identification and quantification were completed, the protein table was imported into Perseus 1.5.2.6 software for the copy number calculation using the “proteomic ruler.”^13,14^

### Gene Ontology Functional Enrichment Analysis

A functional annotation analysis of iPSC-derived CEC genes was completed using Visual Annotation Display (VLAD; ver. 1.6.0), a web-based tool from the Mouse Genome Informatics.^15^ The VLAD tool performs the statistical analysis to test the enrichment of gene ontology (GO) terms based on their annotations to gene function.^15^ A complete set of human genes was used as reference annotation data set, and the ontological terms annotated with the evidence code ND (no biological data) were excluded from the enrichment analysis. The statistically significant enriched terms were sorted based on their corrected *P* value (≤0.01) calculated using multiple testing and positive false-discovery rate for each term.

## Results

### Generation and Validation of Human iPSCs

A 10 mL blood aliquot was obtained from a healthy 67-year-old male with no history of corneal endothelial dystrophy. PBMCs were isolated from a 10 mL blood aliquot by density gradient centrifugation and preserved in liquid nitrogen until further use. The PBMCs were reprogrammed with the integration-free Sendai virus gene delivery method by expressing pluripotent factors (*c-MYC, KLF4, OCT3/4,* and *SOX2*) according to manufacturer's instructions. Embryonic stem cell-like putative iPSC colonies were selected and expanded for characterization (data not shown). The iPSC colonies were evaluated by qRT-PCR for the expression of pluripotent markers, which revealed expression levels of pluripotent markers *NANOG*, *OCT4*, *SOX2*, and *TRA-1-60* in PBMC-originated, iPSCs comparable to hESCs (data not shown). The PBMC-originated, iPSCs were also assessed for the protein expression of pluripotent markers (SSEA4 and TRA-1-60) by flow cytometry. The flow cytometry analysis demonstrated that PBMC-derived iPSCs were positive for SSEA4 (94.56% ± 2.43%) and TRA-1-60 (89.36% ± 1.57%) (data not shown).

### Differentiation of iPSCs Into CECs

**Figure 1:**
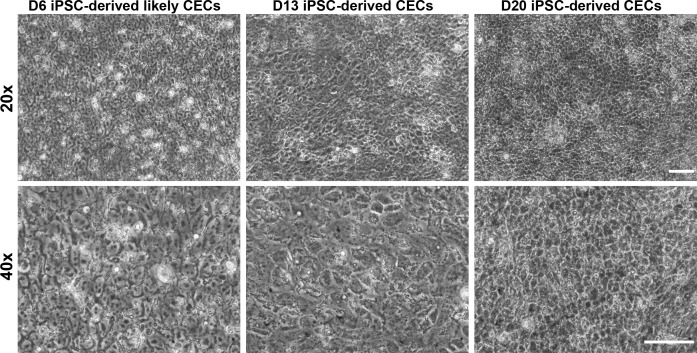
Generation of CECs from human PBMC-originated, iPSCs. Phase contrast microscopy at various magnifications and time points during CEC differentiation illustrating likely CECs at day 6 (D6) and CECs exhibiting CEC-like hexagonal/polygonal morphology at days 13 (D13) and 20 (D20). Note: The images are of 20× and 40× magnifications and the scale bars represent 50 μm.

McCabe and colleagues^9^ recently reported generation of CECs from hESCs. This procedure is based on the differentiation of multipotent NCCs into CECs. We adopted a 20-day procedure modifying the protocol reported by McCabe and colleagues^9^ to generate PBMC-originated, iPSC-derived CECs. We initially characterized the differentiation process by examining the morphology of the differentiating CECs. On day 6, iPSCs differentiated into NCCs, as evident by the expression of neural crest markers (i.e., *NGFR* and *SOX10*) (Supplementary Fig. S1a). On day 7, the prospective CECs were treated with cell dissociation buffer and transferred to a new Matrigel-coated plate. We observed that a transfer to a new Matrigel plate on day 7 is critical for differentiation of iPSCs into CECs. The morphologic examination on day 13 suggested that differentiating CECs have a hexagonal/polygonal appearance (Fig. 1). On day 20, hexagonal/polygonal CECs were tightly packed, having few progenitor-like colonies (Fig. 1; Supplementary Fig. S1b). It is worth noting that the progenitor-like colonies disappeared with extended culture.

### Gene Expression Analysis of Differentiating CECs

**Figure 2:**
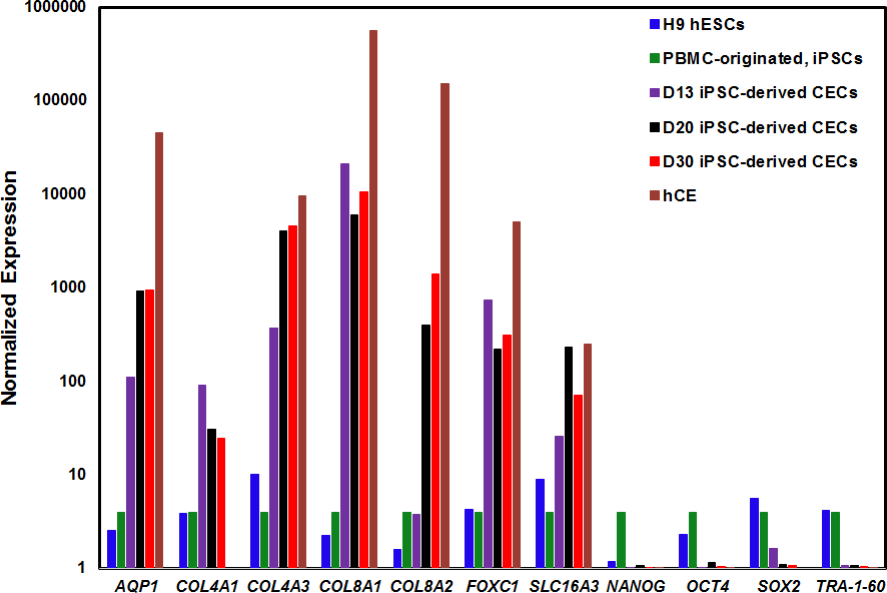
Gene expression analysis of CEC- and pluripotency-associated markers during differentiation of human PBMC-originated, iPSCs into CECs. The expression level of seven CE-associated markers (AQP1, COL4A1, COL4A3, COL8A1, COL8A2, FOXC1, SLC16A3) and four pluripotent markers (NANOG, OCT4, SOX2, TRA-1-60) were analyzed by qRT-PCR in H9 hESCs, PBMC-originated iPSCs, iPSC-derived CECs on days 13 (D13), 20 (D20), and 30 (D30), and hCE. Note: Expression of all markers are normalized against GAPDH, and all values are relative to the respective expression of PBMC-originated, iPSCs.

To confirm the cardinal features of CECs, we performed qRT-PCR analysis for pluripotent, neural crest, and CE-associated markers at different time points during differentiation procedure. We investigated the expression of the pluripotent markers (mentioned above) and CE-associated markers (i.e., *AQP1, COL4A1, COL4A3, COL8A1, COL8A2, FOXC1,* and *SLC16A3*) at multiple time points (day 4, day 13, day 20, and day 30) during differentiation of iPSCs into CECs. On day 13, the expression of CE-associated markers had increased with a concurrent decrease in the expression of pluripotent markers (Fig. 2), strongly suggesting differentiation of iPSCs into CECs. As shown in Figure 2, on day 20, the CECs express *AQP1, COL4A3, COL8A2,* and *SLC16A3,* indicative of CEC maturation. It is of interest that the expression of *COL8A2,* which remained minimal on day 4 and day 13, increased substantially at day 20. The elevated expression of *COL8A1* and *COL8A2* on day 20 is consistent with the previously published report.^9^

The CE-associated markers on day 20 and day 30 revealed a similar expression pattern, except for *COL8A2* and *SLC16A3,* suggesting the differentiating CECs at day 20 have matured to express CE-associated proteins (Fig. 2). It is worth noting that the key pluripotent markers (*NANOG*, *OCT4*, and *TRA-1-60*) exhibited extremely low expression at day 13, and residual expression was observed at day 20 of the differentiation process (Fig. 2).

### Validation of Cryopreserved iPSC-Derived CECs by qRT-PCR and Immunocytochemistry

The differentiated CECs at day 20 were cryopreserved in liquid nitrogen, and a week later the CECs were revived and cultured until they reached confluence. We first evaluated the effect of cryopreservation by phase contrast microscopy. The cryopreserved CECs were tightly adherent, having a hexagonal/polygonal shape (data not shown), suggesting that cryopreservation does not affect the morphology of iPSC-derived CECs. It is worth noting that seeding density of cryopreserved CECs is critical; plating of CECs at low density results in fibroblast-like morphology as CECs expand into the open space.

**Figure 3:**
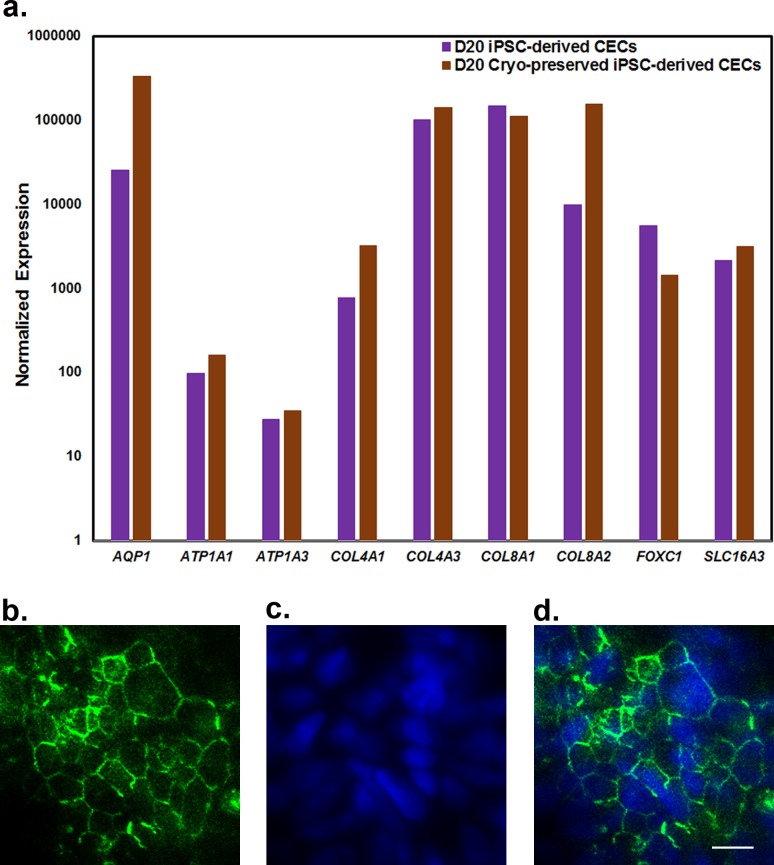
Characterization of PBMC-originated, iPSC-derived CECs following cryopreservation. (a) qRT-PCR analysis of CE-associated markers: AQP1, ATP1A1, ATP1A3, COL4A1, COL4A3, COL8A1, COL8A2, FOXC1, and SLC16A3 on day 20 (D20). (b–d) Immunostaining for zona occludens-1 (ZO-1; a tight-junction protein) exhibiting typical hexagonal/polygonal morphology of CECs. The images are 60× magnification, and the scale bar represents 10 μm.

Next, we investigated the expression of CE-associated markers by qRT-PCR. The qRT-PCR analysis revealed similar expression levels of the CE-associated markers before and after cryopreservation (Fig. 3a), further supporting the notion that cryopreservation does not affect the expression of CE-associated markers in iPSC-derived CECs (Fig. 3a). Finally, we examined the expression of tight-junction protein (ZO-1), N-cadherin, and CE pump function protein (Na^+^/K^+^ATPase α1) by immunocytochemical analysis. The immunostaining of ZO-1, N-cadherin, and Na^+^/K^+^ATPase α1 at the cell boundaries of cryopreserved iPSC-derived CECs illustrating hexagonal/polygonal-like cells confirm the structural integrity of iPSC-derived CECs following cryopreservation (Fig. 3b-d; Supplementary Fig. S2).

### Mass Spectrometry-Based Proteome Sequencing

We performed mass spectrometry–based label-free quantitative proteome profiling to understand the molecular composition of iPSC-derived CECs at day 20. The iPSC-derived CECs from two 35-mm culture dishes were pooled together for proteome profiling. The proteome sequencing generated a total of 463,737 MS/MS count (also referred as PSM), yielding 162,795 total peptides, including 129,750 unique peptides in iPSC-derived CECs (Supplementary Table S1). In total, we identified 10,575 proteins in the iPSC-derived CEC proteome (Supplementary Table S1). The mass spectrometry data have been deposited to the ProteomeXchange Consortium via the PRIDE partner repository,^16^ with dataset identifier PXD009142.

The human genome (GENCODE Version 26; GRCh38) includes 19,817 protein-coding genes and as such, we have identified ∼53% expression (10,575 proteins) of the protein-coding genes in the iPSC-derived CECs. The iPSC-derived CECs revealed a diverse catalog of proteins, including collagens, solute carriers, gap junction, cell adhesion, and junction-interacting proteins (Supplementary Table S1). It is of importance that we identified 32 different collagen proteins, including COL8A1, COL8A2, and COL4A1, in iPSC-derived CEC proteome (Supplementary Table S1), which is consistent with the notion that collagen proteins are a major component of the Descemets membrane.^17^

Additionally, we identified the expression of 150 plus different solute carrier proteins, including ATP1A1, ATP1A3, SLC16A1, SLC16A3, SLC4A2, SLC4A7, CA2, and CA4 in the iPSC-derived CECs (Supplementary Table S1). We observed high expression of tight-junction proteins, including TJP1, TJP2, and TJP3 in the iPSC-derived CEC proteome, and in total, we identified 20 different tight-junction interacting proteins in the iPSC-derived CEC proteome (Supplementary Table S1). The proteome data also revealed expression of multiple cell adhesion and gap junction proteins, especially CDH2 and GJA1 in iPSC-derived CECs (Supplementary Table S1).

We identified expression of cellular metabolism proteins (ENO1, GAPDH, CA3, LDHA, ALDOA, ATP5B, and ATP5A1) and the transmembrane transporter proteins (SLC2A1, ATP5B, ATP1A1, and ATP5A1). We also identified expression of CA3, LDHA, and C4orf49 in the iPSC-derived CEC proteome (Supplementary Table S1) previously identified in CECs.^18,19^ Recently, Song and colleagues^7^ identified three novel shared CE-associated markers (*TRIT1, HSPB11,* and *CRY1*) using a comparative analysis of hESC-derived CECs, human primary fetal, and adult CEC transcriptomes.^7^ Likewise, we also identified these three CE-associated markers in our iPSC-derived CECs.

**Figure 4:**
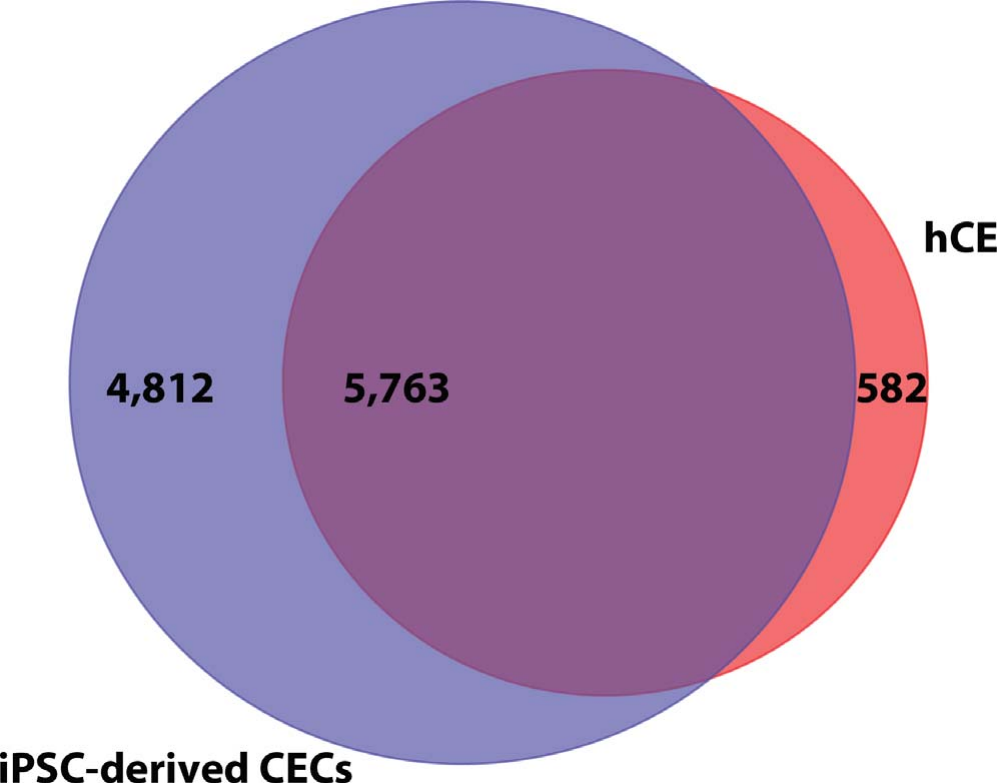
A Venn diagram illustrating the overlap between proteomes of iPSC-derived CECs and hCE. A total of 10,575 and 6345 proteins were identified in iPSC-derived CEC and hCE proteomes, respectively. The blue and red represent proteins identified in iPSC-derived CEC and hCE proteomes, respectively, whereas the overlap represents the common 5763 proteins identified in both iPSC-derived CEC and hCE proteomes.

We performed mass spectrometry–based label-free quantitative proteome profiling of hCE for comparative analysis. A pair of CEs from a 62-year-old female donor (postmortem) was obtained. The donor CE exhibited a uniform cell morphology with a CEC density of 2004 and 2020 cells/mm^2^ from the OD and OS corneas, respectively (Supplementary Fig. S3). The proteome sequencing of hCE generated a total of 114,187 MS/MS count, yielding 57,392 total peptides, including 49,065 unique peptides (Supplementary Table S2). In total, we identified 6345 proteins in the hCE proteome (Supplementary Table S2). Subsequently, the proteomes of iPSC-derived CECs and hCE were compared to identify the shared proteins in both datasets. The analysis revealed the 5763 shared proteins representing 90.82% similarity among both proteomes (Fig. 4).

Finally, we performed the gene ontologies-based functional enrichment analysis of the most abundant iPSC-derived CEC proteins (representing top 35% of total proteins). The gene ontologies of the iPSC-derived CEC proteome were characterized based on molecular function (MF), biological process (BP), and cellular component (CC). The analyses revealed the enrichment of 372, 527, and 2341 significant (*q* value ≤ 0.01) MF, CC, and BP GO terms, respectively (Supplementary Table S3).

## Discussion

Here we report derivation of CEC-like cells from PBMC-originated, iPSCs and characterized the proteome of these iPSC-derived CECs through mass spectrometry–based protein sequencing. Additionally, we examined the effects of cryopreservation on iPSC-derived CECs, which suggested that cryopreservation does not affect the structural integrity of the iPSC-derived CECs. Finally, we investigated the proteome of hCE and performed a comparative analysis of iPSC-derived CECs and hCE that revealed 90.82% similarity among both proteomes.

In the present study, we generated four different iPSC clones and examined each of them for their pluripotency and their potential to differentiate into CECs (data not shown). However, to remain consistent, we selected one clone for generation of iPSC-derived CECs and all subsequent analyses, including mass spectrometry–based proteome sequencing.

We adopted a 20-day procedure modifying the protocol reported by McCabe and colleagues.^9^ McCabe and colleagues reported the generation of CECs by differentiating hESCs through NCCs to exclude the possibility of undesired pluripotent stem cells. We examined differentiating CECs for the expression of neural crest markers at day 6 and identified expression consistent with the previously published report.^9^ We chose iPSC-derived CECs at day 20, as no difference was observed among the expression level of CE-associated markers at day 20 and day 30. The high expression of CE-associated markers and, more importantly, the lack of expression of pluripotency markers strongly support differentiation of iPSCs into the CEC lineage.

In the last few years, several groups have reported transcriptome analysis of cultured CECs, hESC-derived CECs, and the CE.^7,20,21^ Previously, McCabe and colleagues^9^ performed microarray analysis and identified 96% similarity at gene level between hESC-derived CECs and primary human adult CECs.^9^ In the present study, a comparative analysis of iPSC-derived CECs and hCE proteome revealed 90.82% similarity. It is worth noting that we identified 10,575 proteins in the iPSC-derived CEC proteome, whereas only 6345 proteins were identified in the hCE proteome. The discrepancy in the number of proteins identified in these two proteomes stems from the fact that only 210 μg protein was used for hCE proteome profiling compared to 2600 μg of iPSC-derived CEC protein.

It is also worth noting that the iPSC-derived CEC proteome does not contain corneal epithelium-associated markers, that is, KRT3 and KRT12, vascular endothelial markers vWF and PECAM1 (Supplementary Table S1). Moreover, the pluripotency markers NANOG, OCT4, and KLF4 were also absent in the iPSC-derived CEC proteome (Supplementary Table S1).

To the best of our knowledge, we are the first to report generation of peripheral blood originated, iPSC-derived CECs and catalog the proteome of these CECs to understand their molecular composition. We are currently examining the functional ability of iPSC-derived CECs to form barriers, a cardinal characteristic of CE, exploiting transendothelial electrical resistance assays as reported previously.^22^ Future preclinical trial investigations involving transplantation of iPSC-derived CECs in animal models will further elucidate the functional characteristics and the therapeutic potential of these iPSC-derived CECs.

In conclusion, we report generation of iPSC-derived CECs through the NCC lineage using peripheral blood as a donor source with an overall goal to maximize the availability of these vital cells for treating the corneal endothelial disease as an alternative to donor corneas for CE transplantations and other applications.

## Supplementary Material

Supplement 1

Supplement 2

Supplement 3

Supplement 4
