# Supplementary material for: Generation and Proteome Profiling of PBMC-Originated, iPSC-Derived Corneal Endothelial Cells
Source: Invest Ophthalmol Vis Sci. 2018 May;59(6):2437–44. doi: 10.1167/iovs.17-22927 (PMC5957521; doi:10.1167/iovs.17-22927)
Supplement: Supplement 4 [file iovs-59-05-08_s04.pdf]

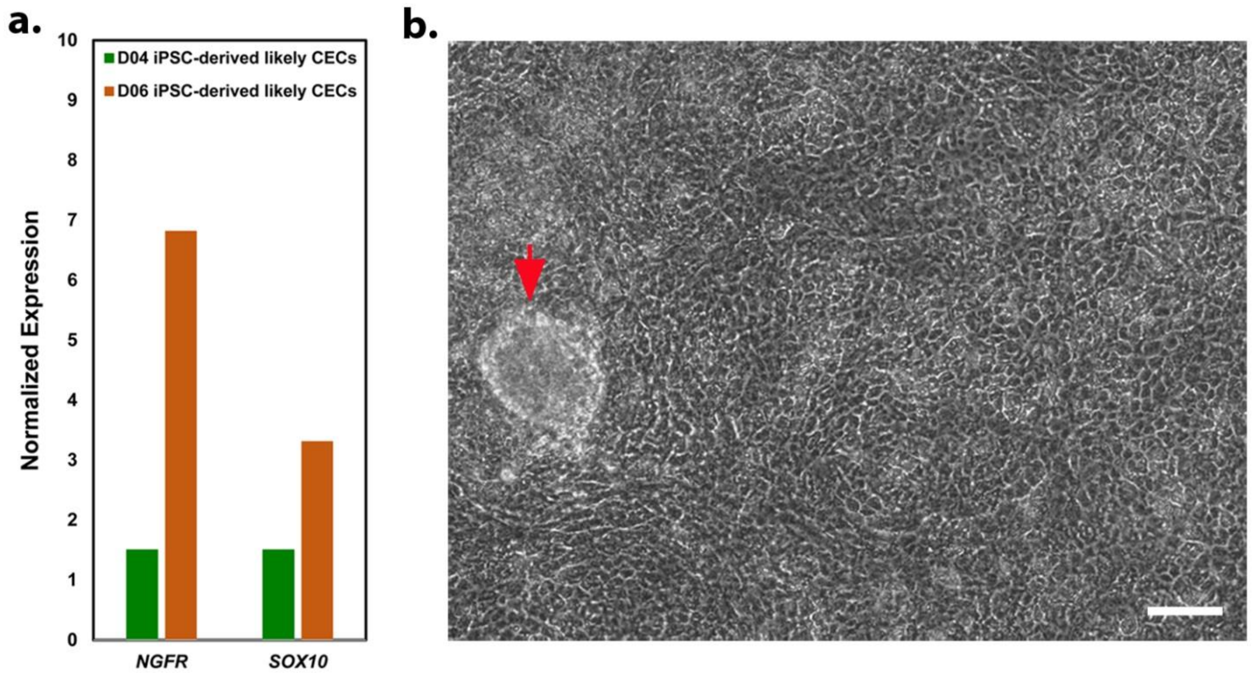

Supplementary Figure 1: Differentiation of human peripheral blood mononuclear cell (PBMC)-originated, induced pluripotent stem cell (iPSC)-derived corneal endothelial cells (CECs). **a.** Gene expression analysis of two neural crest associated markers, *NGFR, and SOX10* were analyzed by quantitative real-time PCR (qRT-PCR) on days 4 (D04) and 6 (D06) of CECs differentiation. **b.** Phase contrast microscopy of CECs differentiation exhibiting progenitor-like colony (pointed by the arrow) at day 20. **Note:** The image is of 10× magnification and the scale bar represents 50 μm.

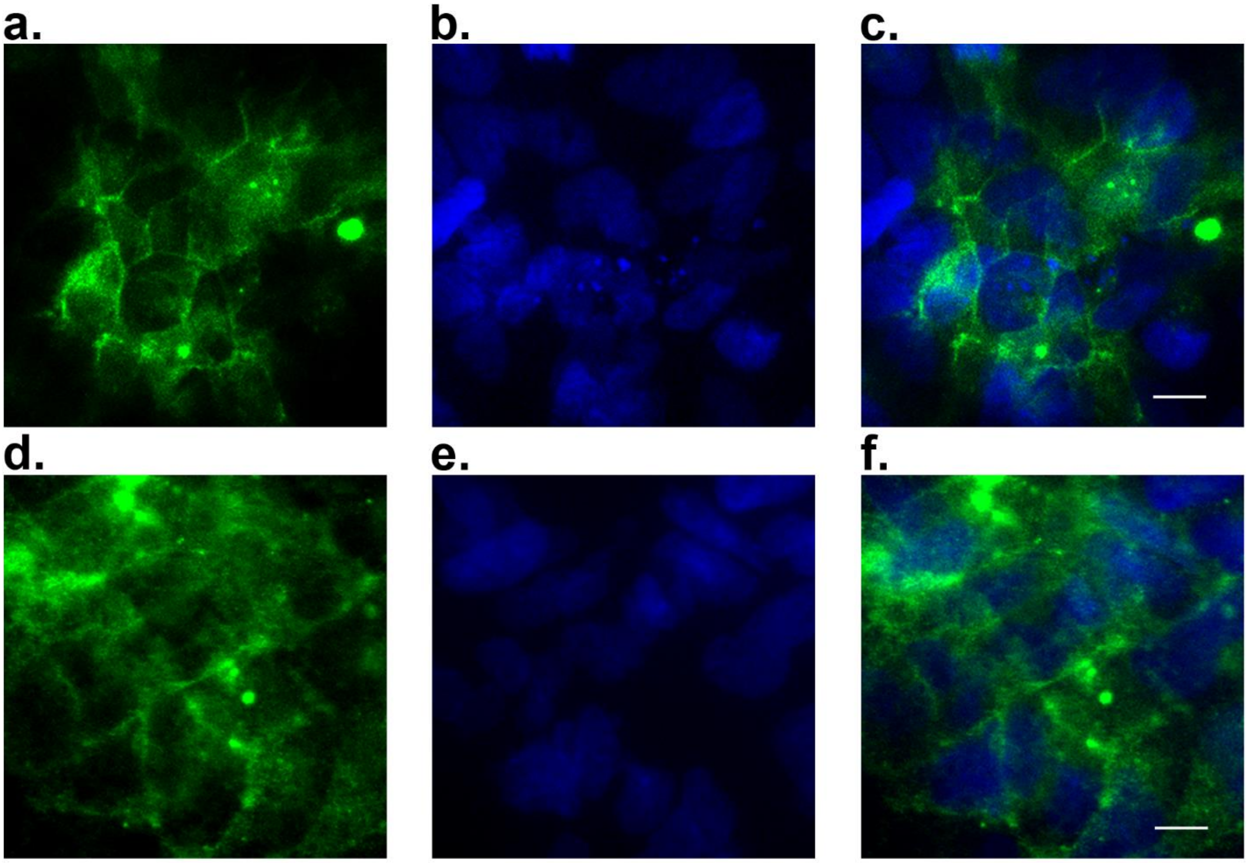

Supplementary Figure 2: Characterization of cryopreserved corneal endothelial cells (CECs) by immunocytochemistry. **a-c.** Immunostaining for N-cadherin exhibiting typical hexagonal/polygonal morphology of CECs. **d-f.** Immunostaining for Na^+/K^+ATPase α1. Cell nuclei were counterstained with DAPI. **Note:** The images are of 60× magnification and the scale bars represent 10 μm.

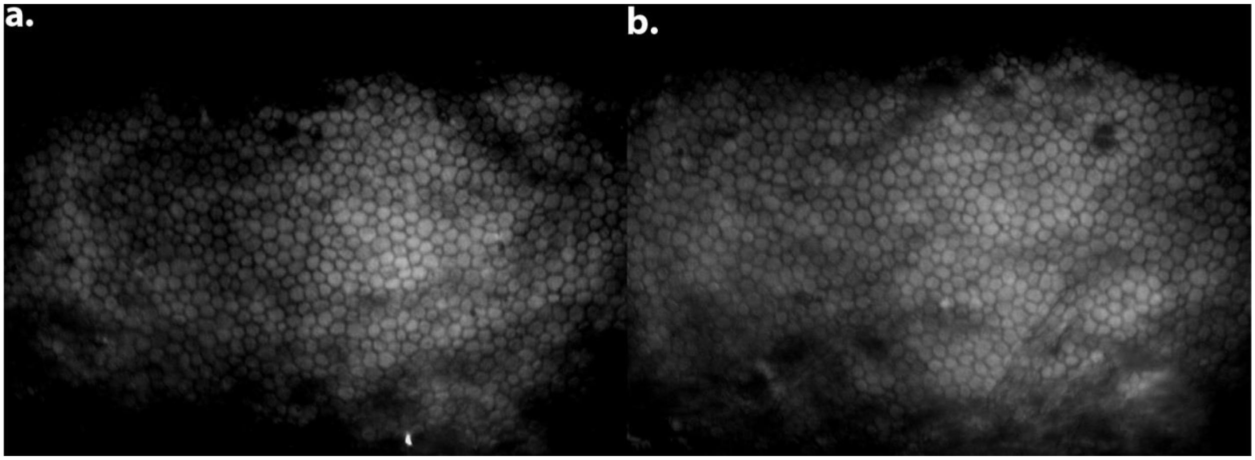

Supplementary Figure 3: Specular microscopy images of the human corneal endothelium (hCE) tissues obtained from a 62-year-old Caucasian female donor (postmortem) used for mass spectrometry-based label-free quantitative proteomics. OD (oculus dextrus: right eye) and OS (oculus sinister: left eye) are marked a and b, respectively.

Supplementary Table 1: A complete list of proteins identified in human peripheral blood mononuclear cell (PBMC)-originated, induced pluripotent stem cell (iPSC)-derived corneal endothelial cell (CEC) proteome at day 20.

Supplementary Table 2: A complete list of proteins identified in the mass spectrometry-based protein sequencing of human corneal endothelium (hCE).

Supplementary Table 3: A complete list of biological process, cellular component and molecular function gene ontology (GO) terms associated with proteins identified in human peripheral blood mononuclear cell (PBMC)-originated, induced pluripotent stem cell (iPSC)-derived corneal endothelial cells (CECs).
